# MicroRNA Expression Profiling Identifies Activated B Cell Status in Chronic Lymphocytic Leukemia Cells

**DOI:** 10.1371/journal.pone.0016956

**Published:** 2011-03-08

**Authors:** Shuqiang Li, Howell F. Moffett, Jun Lu, Lillian Werner, Hao Zhang, Jerome Ritz, Donna Neuberg, Kai W. Wucherpfennig, Jennifer R. Brown, Carl D. Novina

**Affiliations:** 1 Department of Cancer Immunology and AIDS, Dana-Farber Cancer Institute, Boston, Massachusetts, United States of America; 2 Division of Medical Sciences, Harvard Medical School, Boston, Massachusetts, United States of America; 3 Broad Institute of Harvard and Massachusetts Institute of Technology, Cambridge, Massachusetts, United States of America; 4 Department of Biostatistics and Computational Biology, Dana-Farber Cancer Institute, Boston, Massachusetts, United States of America; 5 Department of Medical Oncology, Dana-Farber Cancer Institute, Boston, Massachusetts, United States of America; 6 Department of Medicine, Harvard Medical School, Boston, Massachusetts, United States of America; 7 Department of Neurology, Harvard Medical School, Boston, Massachusetts, United States of America; 8 Department of Pathology, Harvard Medical School, Boston, Massachusetts, United States of America; Cleveland Clinic, United States of America

## Abstract

Chronic lymphocytic leukemia (CLL) is thought to be a disease of resting lymphocytes. However, recent data suggest that CLL cells may more closely resemble activated B cells. Using microRNA (miRNA) expression profiling of highly-enriched CLL cells from 38 patients and 9 untransformed B cells from normal donors before acute CpG activation and 5 matched B cells after acute CpG activation, we demonstrate an activated B cell status for CLL. Gene set enrichment analysis (GSEA) identified statistically-significant similarities in miRNA expression between activated B cells and CLL cells including upregulation of miR-34a, miR-155, and miR-342-3p and downregulation of miR-103, miR-181a and miR-181b. Additionally, decreased levels of two CLL signature miRNAs miR-29c and miR-223 are associated with ZAP70^+^ and IgV_H_ unmutated status and with shorter time to first therapy. These data indicate an activated B cell status for CLL cells and suggest that the direction of change of individual miRNAs may predict clinical course in CLL.

## Introduction

CLL is the most common form of adult leukemia in the western world accounting for approximately 30% of all leukemias in Caucasians. Contrary to its earlier description as a relatively homogeneous disease, CLL more recently has been viewed as a heterogeneous disease with variable clinical course that correlates with several biologic markers of prognosis [1] The most clinically significant prognostic markers are cytogenetics determined by fluorescence *in situ* hybridization (FISH) and IgV_H_ followed by ZAP70 status. Patients with CLL demonstrating deletion of 11q or 17p, high expression of ZAP70 or CD38, or relative absence of V region somatic hypermutation have markers that indicate more aggressive disease.

CLL is characterized as a disease of mature B cells. CLL cells typically express an anergic B cell receptor (BCR) and demonstrate dysregulated apoptotic programs. mRNA expression profiling has been used to classify CLL [2],[3],[4],[5]. Though it is not generally considered a disease of activated B cells, mRNA expression profiling in one study characterized CLL cells as similar to activated B cells [2] and in another as similar to memory B cells [3]. In normal B cells, the nuclear translocation of NF-κB is associated with B cell activation. Constitutive nuclear localization of NF-AT (nuclear factor of activated T cells) and NF-κB2/p52 characterizes CLL cells [6], suggesting an activated B cell state. Moreover, CLL cells demonstrate higher NF-κB DNA binding activity than untransformed B cells, the RelA subunit of NF-κB has been shown to be associated with clinical disease progression, and RelA binding activity is inversely correlated with apoptosis in CLL cells [7]. Recently, CLL cells were shown to express activated cell surface markers and intracellular phenotypes [8].

CLL has also been classified by miRNA expression profiling [9], [10], [11], [12], [13], [14]. Interestingly, none of these miRNA expression profiles for CLL are identical. Dysregulation of particular miRNAs in some CLL signatures have been implicated in the CLL cell apoptotic defect. For example, downregulated miR-15a and miR-16-1 fail to repress Bcl-2 [15] and downregulated miR-29 fails to repress Mcl-1 [16].

Using miRNA expression profiling, we identified a miRNA signature in untransformed B cells induced soon after activation. This activated B cell miRNA signature is also present in CLL cells indicating an activated B cell phenotype for CLL. Our data imply that individual miRNAs involved in B cell activation may participate in the B cell transformation process and could be targets for therapeutic gene silencing in CLL.

## Materials and Methods

### Control B cell donor and CLL cell patient characteristics

Cells from 38 CLL patients were assessed in this study. All patients were enrolled on a tissue banking protocol, #99-224, prior to sample collection. This tissue banking protocol was approved by the Dana-Farber Cancer Institute (DFCI) Institutional Review Board, and informed consent was obtained from all CLL patients prior to sample collection. These patients had white blood counts (WBC) between 15.7×10^3^ and 265.6×10^3^ cells per microliter of blood. 3 of the CLL patients were treated, while the other 35 patients were untreated. Control blood samples were from healthy donors. ZAP-70 status, IgV_H_ mutation, and genomic aberrations were determined as described in Methods S1. The clinical parameters are summarized in Table S1.

### Control B cell and CLL cell purification

Ten mL of heparinized peripheral blood was subjected to Ficoll-Paque™ PLUS (GE Healthcare, Pittsburgh, PA, USA) density centrifugation. Control B and CLL cells were purified from peripheral blood mononuclear cells (PBMCs) by positive selection with CD19^+^ selection beads as suggested by the manufacturer (StemCell Technologies Inc, Vancouver, Canada), and then were immediately used for RNA isolation or activation treatment.

### Control B cell culture and activation

B cells were cultured in Iscove's Modified Dulbecco's Medium (IMDM) (GIBCO, Carlsbad, CA, USA) supplemented with 10% human AB serum (Mediatech, Inc, Herndon, VA, USA), 1% Penicillin-Streptomycin (GIBCO, Aukland, NZ, USA), 1% HEPES (Mediatech, Inc, Herndon, VA, USA), 1% L-glutamine (Mediatech, Inc, Manassas, VA, USA), 50 µg/mL human transferrin (Sigma, St. Louis, MO, USA) and 5 µg/mL insulin (Sigma, St. Louis, MO, USA). The cells were kept in a 37°C incubator with 5% CO_2_.

For CD40 activation, NIH-3T3 CD40L or NIH-3T3 feeder cells were cultured to confluency and then irradiated for 9 K by Cs-irradiator. Irradiated feeder cells were plated in 6 well plates at 2×10^5^ cells/mL overnight at 37°C. The cells were washed twice gently with PBS and the B cell medium was added. Purified control B cells were co-cultured with the feeder cells at 37°C for 24 hr or 48 hr.

For BCR activation, purified control B cells were adjusted to 1×10^7^ cells in 0.1 M phosphate buffered saline (pH 7.2). Goat anti-human immunoglobulin M (IgM) F(ab')_2_ (Southern Biotechnology Associates, Birmingham, AL, USA) at 10 µg/mL was added to untransformed B cells for 20 min. on ice. These cells were transferred to human B cell medium and cultured for 24 hr or 48 hr at 37°C. For other methods of activation, 5×10^6^ control B cells were plated in 5 mL of medium containing lipopolysaccharide (LPS) (0.5 ng/µL, Alexis, San Diego. CA, USA), CpG (1 µM, Hycult Biotech, Canton, MA, USA, the sequence of the CpG is 5′ tcgtcgttttgtcgttttgtcgtt 3′), or polyI∶C (5 ng/µL, Invivogen, San Diego, CA, USA) at 37°C for 24 hr or 48 hr.

For experiments involving inhibitors for the c-jun NH2-terminal kinase (JNK) or mitogen-activated protein kinase/ERK kinase (MEK), cells was co-cultured with the JNK inhibitor SP600125 (50 µM, A. G. Scientific, Inc., San Diego, CA, USA) or MEK inhibitor PD98059 (50 µM Sigma, St. Louis, MO, USA) for 24 hr before the isolation of RNA for RT-PCR assay.

### FACS analysis

CLL cell purity was tested using anti-CD5-PE and anti-CD19-FITC (BD Pharmingen, San Diego, CA, USA) antibodies. The activation of control B cells was tested using anti-CD69-FITC, anti-CD80-FITC or anti-CD86-FITC (BD Pharmingen, San Diego, CA, USA). These antibodies were incubated on ice with the B cells in the presence of PBS with 2% FBS (GIBCO, Carlsbad, CA, USA) for approximately 30 min., the cells were washed with PBS at 4°C for 10 min., centrifuged for 100 g at 4°C, supernatants were aspirated. The cell pellets were re-suspended in 200 µL of PBS and analyzed by FACSCalibur (Becton Dickinson).

### Total RNA preparation

Total RNA from control B or CLL cells was extracted by Trizol method (Invitrogen, Carlsbad, CA, USA). Dried RNA pellets were re-suspended in appropriate volumes of DEPC/ddH_2_O. RNA was quantitated by O.D._260/280_ using spectrophotometry DU640 (Beckman).

### MiRNA expression profiling

We first profiled 435 miRNAs from 22 CLL samples and 1 control B sample by the Luminex method requiring multiple gel extraction steps as described [17]. To expand our sample size, we then chose 43 miRNAs which were predicted to be differentially expressed in CLL from our previous data, 4 miRNAs from published data, and 3 control miRNAs (listed in Table S2) for large scale profiling with the FlexmiR miRNA labeling kit (Luminex, Austin, TX, USA, as described [18]. In this method, 2.5 µg of total RNA was labeled with biotin, and then it was hybridized with locked nucleic acid (LNA)-modified capture probe coupled with beads. After washing away the unbound RNA samples, streptavidin-phycoerythrin (SAPE) reporter molecules were added to the reaction and then the expression of miRNAs was analyzed on Luminex analyzer.

### Data analysis for miRNA profiling

A mock array was generated by averaging the expression of each miRNA in all samples. Next, a linear correction for each sample to the mock array was performed. The data were analyzed using GenePattern software from Broad Institute. MiR-146b-5p was deleted from the analysis due to abnormally high background bead signals.

### Gene set enrichment analysis

We defined a B cell miRNA activation signature as miRNAs that were significantly altered with a t-test p-value of <0.05 after correction for multiple comparisons in control versus *in vitro* activated B cells. We performed independent GSEA analysis for upregulated and downregulated miRNA sets to test for enrichment of activation signature miRNAs in control B cells versus CLL profiles as described in [19].

### MiRNA-specific RT-PCR assay

Total RNA was treated with DNase I (Ambion, Austin, TX, USA), and cDNA synthesis was carried out using miScript Reverse Transcriptase kit (Qiagen, Hilden, Germany). miRNA-specific RT-PCRs were performed using the miScript primer assay according to the manufacturer's protocol (Qiagen, Hilden, Germany). The miRNA specific primers and the internal control primers were obtained from Qiagen (Hilden, Germany). The relative expression of specific miRNAs was calculated by the ΔΔCt method. A randomly chosen subset of the CLL cell RNAs samples used for Luminex bead-based miRNA expression profiling were used for validation by RT-PCR-based miRNA expression profiling.

### Statistical methods

Associations between miRNA expression and clinical characteristics were assessed using the Fisher exact test for binary variables, and the Kruskal-Wallis test for variables with three or more categories. Time to first therapy was calculated as time from initial diagnosis to first therapy; patients not yet treated were censored at date last known alive. Time to first therapy was estimated using the method of Kaplan and Meier; the log rank test was used to assess associations with time to first therapy. Recursive partitioning was used to identify an optimal binary split for each miRNA individually, using the *rpart* package in R. *P*-values from recursive partitioning are not adjusted for the optimization of the method. Two-sided *p*-values are not adjusted to reflect multiple comparisons; *q*-values reflect adjustment for multiple comparisons using the false discovery rate of Benjamini and Hochberg, as implemented in the *q*-value package in R. A false discovery rate of 0.10 or smaller was the criterion for reporting significant differences in both clinical features and time to first therapy in this study.

## Results

### Determinants of a CLL-specific miRNA expression signature

CLL cells are characterized by expression of both CD5 and CD19 cell surface markers [20]. CD19 bead positive selection was used to purify CLL cells and greater than 97% CLL cell purity was verified by FACS analysis of CD5 and CD19 expression for all samples used in this study (Figure S1). We profiled miRNA expression across 38 highly purified CLL patient samples, 9 control B cell samples and 5 activated B samples by CpG using the Luminex method [18]. Unsupervised hierarchical analysis clustered CLL samples based upon miRNA expression (Figure S2). Comparative marker selective view alignment analysis in CLL samples compared with control B samples identified a CLL-specific miRNA signature consisting of upregulation of let-7g, miR-26a, miR-29a, miR-29b, miR-29c, miR-101, miR-150, and miR-155, and downregulation of miR-23a, miR-24, and miR-27b, miR-181a, miR-181b, and miR-223 (Figure S3).

Though most B cells are CD5-CD19+, specific subsets of B cells (e.g. peritoneal, tonsillar) are CD5+CD19+. To verify that miRNA expression profiling discerned differences between normal CD5-CD19+ B cells and CD5+CD19+CLL cells rather than between normal CD5-CD19+ and CD5+CD19+ B cells, control B cells from 4 different control donors were sorted into CD5- and CD5+ populations and RT-PCR analysis was performed. Our data indicate that miR-181a and miR-181b were downregulated in 6 CLL samples relative to both CD5- and CD5+ control B cell populations. Similarly, miR-29a, miR-150, and miR-155 were upregulated in CLL relative to both CD5- and CD5+ control B cell populations (Figure S4). These data indicate that CD5+ expression status does not affect the interpretation of the changes in miRNA expression in these studies. Notably, CD19 positive purification does not alter miRNA expression as assessed by RT-PCRs (Figure S5).

### miRNA alterations identify an activated B cell phenotype in CLL cells

Several characteristic miRNAs that have altered expression in CLL relative to untransformed B cells are also important for lymphocyte activation (e.g. miR-181a [21], miR-181b [22], miR-150 [23], and miR-155 [24]), inflammatory responses (e.g. miR-155 [25], [26]), and B lymphoproliferative disorders (eg. miR-155 [27], [28]). We thus hypothesized that CLL cells may derive from partially activated B cells.

**Figure 1:**
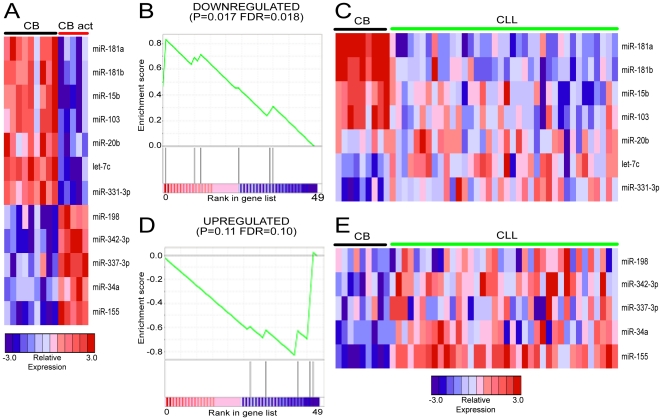
GSEA reveals a B cell activation miRNA signature in CLL. A. Heatmap of B cell activation signature miRNAs comparing expression in unstimulated control B (CB) cells and CpG activated B cells (CB act). B. GSEA enrichment profile of downregulated B cell activation signature miRNAs in control B cells versus CLL cells. C. Heatmap of downregulated B cell activation signature miRNA expression in control B cells and CLL cells. D. GSEA enrichment profile of upregulated B cell activation signature miRNAs in control B cells versus CLL cells. E. Heatmap of upregulated B cell activation signature miRNA expression in control B cells and CLL cells.

To systematically compare miRNA expression in CLL to the miRNA changes induced by B cell activation, we identified sets of miRNAs significantly (*p*<0.05) upregulated or downregulated after untransformed B cell activation by CpG. The upregulated miRNAs were miR-34a, miR-198, miR-155, miR-337-3p and miR-342-3p and the downregulated miRNAs were let-7c, miR-15b, miR-20b, miR-103, miR-181a, miR-181b, and miR-331-3p (Figure 1A). We then performed GSEA for these upregulated and downregulated miRNAs in CLL versus untransformed B cells (Figure 1B and 1D). Four out of seven downregulated miRNAs (miR-15b, miR-103, miR-181a, and miR-181b) were expressed at lower levels in CLL, and five out of five upregulated miRNAs (miR-34a, miR-155, miR-198, miR-337-3p and miR-342-3p) were expressed at higher levels in CLL compared to untransformed B cells (Figure 1C and 1E).

**Figure 2:**
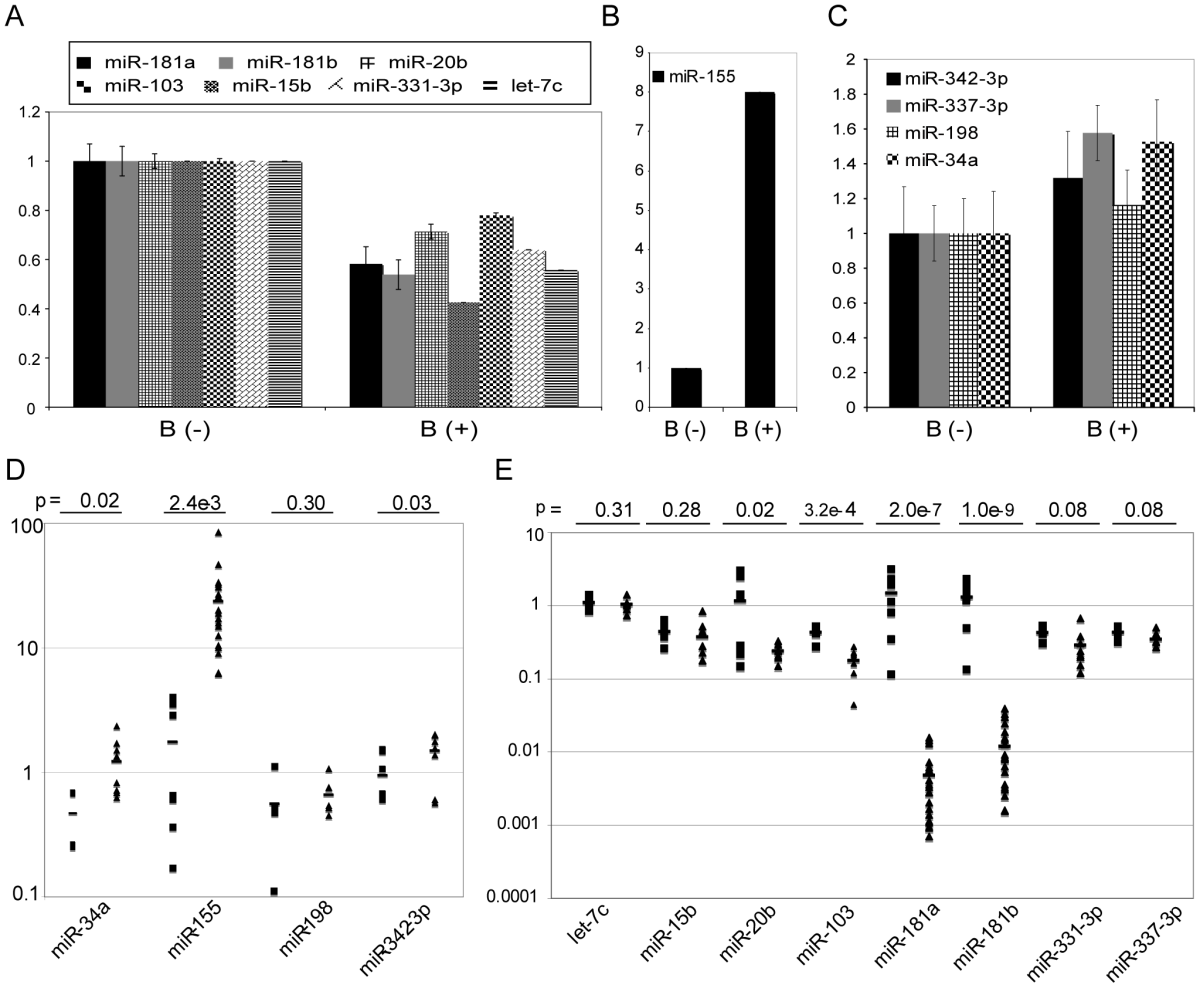
Validation of altered miRNA expression identified by GSEA. miRNA-specific RT-PCR independently confirms altered expression of GSEA-identified miRNAs in B cells activated with CpG. A. miR-181a, miR-181b, miR-20b, miR-103, miR-15b, miR-331-3p, and let-7c. B. miR-155. C. miR-342-3p, miR-337-3p, miR-198, and miR-34a. For (A, B, C), B (-): control B cell samples. B (+): corresponding activated B samples by CpG. Error bars indicate the standard deviation of 3 independent samples. D, E. miRNA-specific RT-PCR confirms the differential expression of subsets of GSEA-identified miRNAs in CLL relative to control B cells. Upregulated miRNAs are miR-34a, miR-155, miR-198 and miR-342-3p. Downregulated miRNA expression including let-7c, miR-15b, miR-20b, miR-103, miR-181a, miR-181b, miR-331-3p and miR-337-3p. Between 4 to 8 control unstimulated B cell samples (square) and between 6 to 21 CLL patient samples (triangle) were used. The bar indicates the average expression in each group. *p*-value for the analysis calculated by t-test is shown. miRNA expression was normalized by snoRNA-RNU44 expression. Relative miRNA expression was calculated by ΔΔCt method and is indicated on the Y-axis.

Next, we used miRNA-specific RT-PCR to confirm the expression of these signature miRNAs in 3 control B samples and 3 activated B samples (different from the samples used in the miRNA profiling) after CpG activation (Figure 2A, 2B, and 2C) and in control B samples and at least 4 CLL patient samples per miRNA, (Figure 2D and 2E). Using RT-PCR, we independently confirmed these miRNA expression patterns in control B and activated B cells (Figure 2A and 2B). However, in CLL cells, miR-337-3p demonstrated an opposite trend to the one identified in GSEA of miRNA expression profiling (Figure 1E). Moreover, miR-15b and miR-198 demonstrated a similar trend in as in GSEA of miRNA expression profiling (Figure 1C and 1E) though the p value was not statistically significant (Figure 2D and 2E). Variations detected between Luminex bead-based profiling and RT-PCR may be due to the heterogeneity of CLL as we used a subset of CLL samples for the qPCR confirmation.

To mechanistically link altered miRNA expression in CLL with altered expression of miRNAs observed in B cell activation, we carefully examined the expression of one miRNA (miR-155) whose expression is increased in CLL and in activated B cells. The miR-155 gene is activated upon B cell stimulation and contains binding sites for the AP-1 transcription factor. B cell activation stimulates the JNK pathway, increases the levels of phospho-ERK, and then activates AP-1 [29]. Treatment of CpG activated B cells and CLL cells with either JNK or MEK inhibitor decreased the expression of miR-155 (Figure S6A and S6B). These data indicate common signaling pathways affect altered miRNA expression observed in activated B cells and CLL cells.

**Figure 3:**
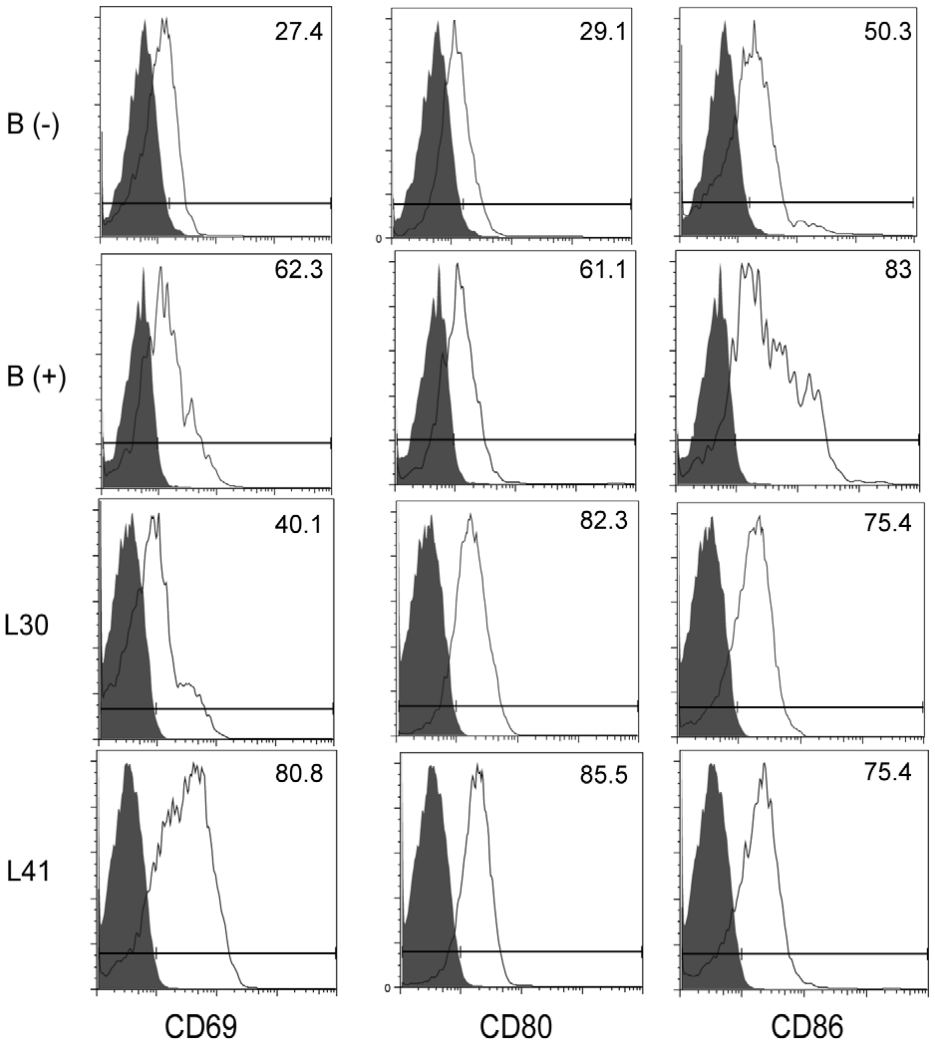
CLL cells express elevated levels of B cell activation markers. Unstimulated donor B cells, anti-IgM-activated donor B cells, or CLL cells were stained for CD69, CD80 or CD86 levels by FACS analysis. Grey filled curve shows the isotype control, open curve shows the specific antibody staining. One representative unactivated donor B cell sample (B-), one anti-IgM-activated B cell sample (B+) and two CLL samples (L30 and L41) are shown. An expanded analysis of donor and patient samples is shown in Table S3.

To confirm the activation phenotype indicated by miRNA expression profiling, we performed FACS analysis of naïve B cells, activated B cells, and CLL cells using B cell activation markers CD69, CD80, and CD86 (Figure 3 and Table S3). Our data demonstrate that CLL cells express CD69 and CD80 at levels that approximate the levels observed in activated B cells and that CLL cells express CD86 at levels intermediate between naïve and stimulated B cells. These data indicate that CLL cells have similar gene expression patterns as activated B cells.

To independently verify that miRNA changes observed in CLL cells are characteristic of an activated B cell status, we purified B cells from healthy donors and stimulated these cells with a variety of B cell activators including anti-IgM and CD40L (BCR and T cell-assisted co-stimulatory pathways), and LPS, CpG, or polyI∶C (Toll-like receptor pathways) and examined miRNA expression. Stimulation of highly purified, control B cells was verified by FACS analysis of cell membrane expressed activation markers including CD69, CD80 and CD86 expression (Figure 3 and Table S3).

In addition to the activation signature, additional miRNAs are differently expressed in CLL compared to untransformed B cells. We tested the expression patterns of these miRNAs to determine if they also were altered in untransformed B cells upon activation. For the CLL signature miRNAs, we found that activation of control B cells led to reduced miR-23a, miR-23b, miR-24, miR-27b, miR-181a, miR-181b, and miR-223 (all downregulated in the CLL signature) and increased miR-155 (upregulated in the CLL signature), regardless of the mechanism of B cell activation (Figure S7 and summarized in Table 1). Most miRNAs demonstrated consistently altered expression regardless of the mechanism of B cell activation though the expression of the miR-29 family varied depending upon B cell activation. Whereas anti-IgM and CD40L upregulated miR-29a (Figure S7A and S7B), LPS, CpG, or polyI∶C downregulated miR-29a (Figure S7C). In contrast, anti-IgM and CD40L downregulated miR-29b and miR-29c (Figure S7A and S7B) but LPS, CpG, or polyI∶C upregulated miR-29b and miR-29c (Figure S7C). miR-223 is increased in response to CD40L activation (Figure S7B), while decreased in response to all other stimuli (Figure S7A and S7C). Additionally, the expression of miR-26a was increased in CLL and only in B cells activated with CD40L but did not change in B cells activated with anti-IgM (Figure S7A and S7B). These data suggest that particular CLL signature miRNAs are altered in response to specific B cell stimuli. In contrast, miR-150 was reduced during B cell activation, and upregulated in almost all the CLL samples tested (Table 1). Thus, high expression of miR-150 may be CLL specific.

**Table 1:** Signature miRNA expression comparison between activated B cells and CLL cells relative to control B cells.

miRNA list	Activation			CLL
	IgM/CD40L	TLRs		
let-7g	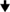	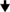	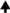	
miR-23a	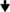	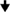	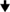	
miR-23b	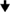	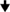	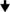	
miR-24	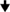	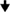	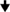	
miR-26a	-/ 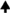	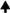	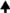	
miR-27b	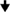	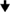	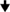	
miR-29a	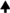	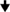	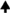	
miR-29b	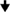	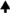	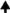	
miR-29c	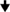	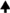	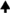	
miR-101	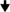	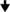	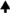	
miR-150	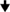	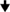	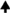	
miR-155	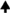	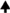	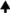	
miR-181a	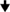	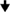	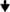	
miR-181b	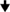	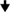	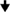	
miR-223	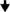 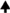	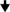	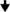	

Up and down arrows indicate upregulation or downregulation of specific miRNAs, respectively, when compared to untransformed, control B cells. A parallel line indicates no change when compared to untransformed, control B cells. miR-223, miR-29a, miR-29b and miR-29c are upregulated in response to certain B cell activators and downregulated in response to other B cell activators. miR-26a is unaffected by IgM stimulation, but upregulated in response to other B cell activators.

### Signature miRNA alterations are associated with clinical features and time to first therapy

Previous reports suggest that altered miRNA expression has prognostic value [30], [31], [32]. ZAP70 and IgV_H_ mutational status are strongly associated with clinical outcome for CLL patients. Thus, comparative marker alignments were performed to identify miRNAs clustered by ZAP70 and IgV_H_ status (Table S4A and S4B). Out of 38 patients, we examined 14 ZAP70^+^ patients and 14 IgV_H_ unmutated patients (Table S1). We found that miR-150 was upregulated in ZAP70^+^ patients whereas miR-29c and miR-223 were upregulated in both ZAP70^−^ and in IgV_H_ mutated patients suggesting that decreased levels of miR-150 and increased levels of miR-29c and miR-223 may be associated with better clinical outcomes. Our data do not indicate a statistically-significant correlation between miR-29c and miR-223 with any established cytogenetic abnormalities used to distinguish CLL patient sub-populations, such as 17p, 11q and 13q deletion and trisomy 12 (data not shown). Additionally, miR-92a was upregulated in ZAP70^−^ patients and let-7g was upregulated in IgV_H_ mutated patients (Table S4A and S4B).

**Figure 4:**
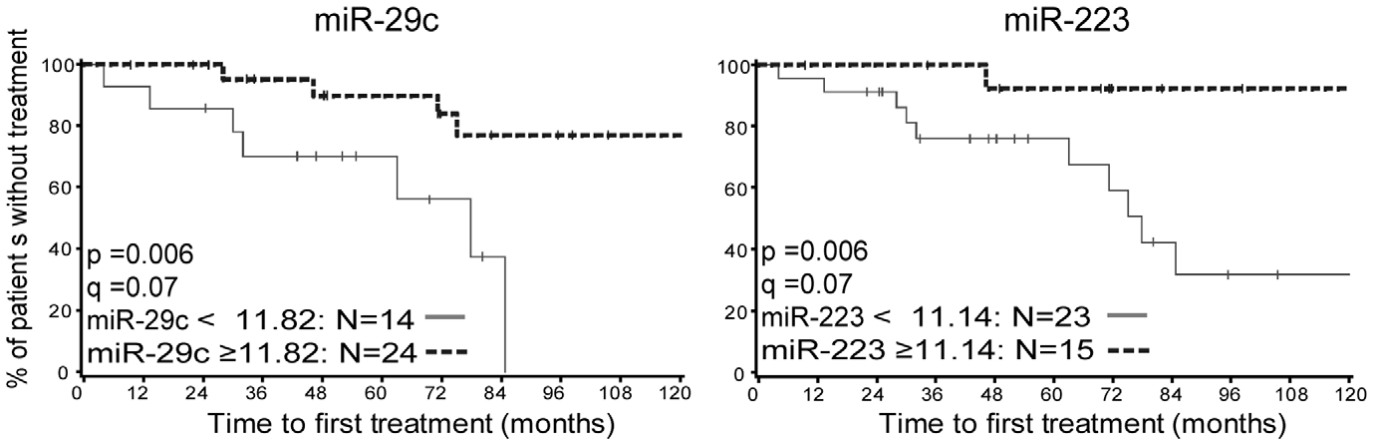
The expression of miR-29c and miR-233 is associated with time to first therapy in CLL. A shorter time from diagnosis to initiation of first therapy is significantly associated with reduced expression of miR-29c and miR-223. A recursive partitioning approach identifies the optimal split for each miRNA: miR-29c expression at 11.82 or lower is associated with shorter time to first therapy, *q* = 0.07. miR-223 expression at a level of 11.14 or lower is associated with shorter time to first therapy, *q* = 0.07.

We also examined miRNA expression across CLL cells for association with time to first therapy in these patients. Using recursive partitioning analysis for finding optimal cut points for miRNA expression, we examined time to first therapy. We identified two miRNAs (miR-29c and miR-223) with *q*-values of 0.07 associated with an optimized binary split (Kaplan-Meier curves and the optimal splits are indicated (Figure 4)). CLL patients with lower expression levels of these miRNAs demonstrated shorter time to first therapy compared to CLL patients who did not demonstrate this pattern of miRNA expression. Decreased expression of these two miRNAs was also associated with ZAP70^+^ and IgV_H_ unmutated patients (Table S4A and S4B) implying poor prognosis.

## Discussion

miRNA expression profiling has been used to identify signatures that classify CLL [10], [11], [12], [33]. Interestingly, none of these published signatures are identical (Table S5). The reasons for the different miRNA signatures are unknown. Different methods and different miRNA analysis platforms may partly explain the different signatures identified by each of these groups. Calin et al. profiled 94 CLL patient samples using a miRNA microarray but did not purify CLL cells to homogeneity [10], [11]. Fulci et al. profiled 56 CLL patient samples by cloning and SYBR-green-based RT-PCR [12]. Zanette et al. profiled miRNA expression in 9 CLL patients and 6 healthy donors using TaqMan-based miRNA assays [13]. However, CLL is a heterogeneous disease and it is possible that different patient populations have unique miRNA expression patterns. Interestingly, Zhang et. al. [14] demonstrated CLL cells are more like memory B cells based on miRNA profiling, our miRNA profiling data showed CLL cells are behaving like activated B cells at an earlier time point.

The uniformity of miRNA expression changes in CLL patient samples relative to B cells is striking (Figure 1 and Figure S3). Though the directionality of changes in expression is consistent for these particular miRNAs, the magnitude of changes varies considerably for these particular miRNAs. Indeed, some miRNAs demonstrate expression variance over several orders of magnitude. This variability in expression of these miRNAs may be related to the heterogeneous clinical course. Notably, nine of the 12 miRNAs changed in the same direction in CLL cells compared to their changes when B cells are activated (Figure 1). Several factors can affect the direction of miRNA expression changes in CLL cells and activated B cells including the timing of altered miRNA expression, type of stimulation, or that certain changes may be important to the pathobiology of CLL. Interestingly, the expression of the two prognostic miRNAs (miR-29C and miR-223) varies in activated B cells depending upon the type of stimulation (Table 1). Interrogating the causes and consequences of altered miRNA expression in CLL cells in common with and distinct from altered miRNA expression in activated B cells will improve our understanding of this disease.

Another striking finding is that certain CLL signature miRNAs demonstrate similar trends in activated B cells regardless of the mechanism of activation (Table 1). Mir-23a, miR-23b, miR-24, miR-27b, miR-155, miR-181a, miR-181b, and miR-223 are all altered consistently with activated B cells. However, certain CLL signature miRNAs are altered consistent with specific B cell activators. For example miR-29b and miR-29c are downregulated with IgM and CD40L activation but are upregulated with TLR activation. Two recent reports examine differential miRNA expression in naïve, germinal center, memory and plasma B cell populations [14], [34]. Interestingly, miR-29c is reduced in germinal center B cells [14] and in centroblasts [34] but is increased in memory B cells [14]. The pattern of miR-29 family member expression may be affected by the duration of B cell activation and/or to multiple B cell activators present in germinal centers proceed through T cell-dependent (e.g. CD40L) and T cell-independent (e.g. anti-IgM) pathways acting simultaneously. Still, certain alterations in miRNA expression are common with published observations such as downregulated miR-181b and miR-223 [34] regardless of the mechanism of activation suggesting that B cell activation may converge on common miRNA-regulated pathways. Importantly, these common miRNAs are also present in the CLL signature reported here.

Recent reports indicate that certain miRNAs identified in this CLL signature have prognostic value in CLL [30], [31], [32] Additionally, CLL patients expressing CD38 tend to have higher expression of activated cellular markers, including CD27, CD62L, CD69 and KI-67, and this activation status is correlated with poor clinical outcomes [35], [36]. Our study integrates these two observations. We found that miRNAs associated with poor clinical outcomes are a part of a larger pattern of miRNA changes consistent with B cell activation. In B cells, miR-29c and miR-223 are both reduced after BCR or co-stimulatory activation. In CLL cells, decreased miR-29c and miR-223 are associated with ZAP70^+^ and IgV_H_ unmutated status (Tables S4A and S4B) and shorter times to first therapy (Figure 4).

We do not have access to sufficient number of fresh samples from additional patients with full clinical annotation to serve as a validation set for these observations. However, these observations are consistent with previous observations that have associated reduced levels of these two miRNAs with ZAP70^+^ and IgV_H_ unmutated status [11], [12], [32], [37]. Moreover, previous studies have associated reduced miR-29c with p53 status and 17p deletion, the worst prognostic group in CLL [30], [31]. We did not identify an association between reduced miR-29c and any cytogenetic abnormalities (data not shown). miR-29c and miR-223 co-vary with other clinical parameters and thus may not have independent prognostic value, but these miRNA changes may have biological function in CLL. For example, reduced levels of miR-29c have been shown to de-repress expression of MCL-1 and TCL-1, known oncogenes implicated in the CLL anti-apoptotic defect [30], [31], [33]. BCR activation led to downregulation of miR-29c and miR-223 (Figure S7A) and ZAP70 is associated with increased BCR signaling in CLL [38]. It is likely that miR-29c and miR-223 are negatively affected by enhanced BCR signaling mediated by ZAP70 [38]. Although the kinase domain of ZAP70 is not required for BCR pathway stimulation in CLL, the scaffolding function of ZAP70 acts as an adaptor that clusters and thereby increases signaling through BCRs in CLL [38], [39]. Thus, downregulation of miR-29c and miR-223 may potentiate BCR signaling and accelerate CLL oncogenesis.

Other miRNA changes consistent with B cell activation may also have an important role in CLL oncogenesis. miR-155 demonstrates an average of 50-fold increase in expression in CLL cells tested compared with control B cells (Figure 2D), is increased in multiple cancers [27], [28], [40], and promotes B cell lymphomagenesis [41]. Conversely, miR-181a and miR-181b demonstrate approximately a 100-fold decrease in expression in virtually all CLL cells tested as compared with control B cells (Figure 2E). PI-3K is a predicted target of miR-181a and miR-181b [42] and the PI3K pathway has been implicated in the apoptotic defect in CLL cells [43]. These data suggest that miR-155 has an oncogenic function whereas miR-181a and miR-181b have a tumor suppressive function in CLL. Analysis of changes in miRNA expression and their target genes during B cell activation and CLL oncogenesis will provide insights into the physiological roles of these miRNAs. Understanding the roles of miRNAs in the dysregulated gene networks that underlie the pathology of CLL could enable the application of miRNA-based therapeutics for this common leukemia.

## Supporting Information

Figure S1 **Purity of CLL sample after CD19 positive selection.** FACS analysis was performed on PBMCs (PBMC) and purified CLL cells (CLL) using anti-human CD19 and anti-human CD5 antibodies. The purity of the CLL cells was shown as the percentage number on the CD5+CD19+population.
(TIF)

Figure S2 **Hierarchical clustering of miRNAs expressed in CLL, control B cell, and activated B cells.** Heatmap of miRNA expression across 38 patient-derived CLL samples, 9 donor B cell samples, and 6 CpG activated donor B cell samples. miRNA expression is hierarchically clustered on the Y-axis and patient-derived CLL samples or control B cell donors are hierarchically clustered on the X-axis. The relative expression of miRNAs is depicted according to the color scale shown on the right. CB#: donor control B sample; CB# act: donor activated B samples by CpG; L#: CLL samples.
(TIF)

Figure S3 **Comparative marker selective view alignment miRNA expression distinguishes miRNA expression between unactivated donor B cells from CLL cells.** Comparative marker selective view alignment distinguishes upregulated miRNA expression in 9 control, unactivated B cell sample (Y-axis, red) and in 38 CLL cell patient samples (X-axis, blue). *Score* (Y-axis) refers to the t-test score indicating the metric correlating gene expression and phenotype. The calculation of t-test score is: (µ1–µ2)/ (s1∧2+s2∧2)∧0.5, where µ1 is the mean of class 1 and µ2 is the mean of class 2, and s1 and s2 are the standard deviation of class 1 and 2. A high score indicates association with the first phenotype (upregulated in control B cells) and a low score indicates association with the second phenotype (upregulated in CLL cells). False Discovery Rate (FDR) is 0.004 for all the miRNAs shown in the diagram.
(TIF)

Figure S4 **miRNA expression profiling in CD5+B cells in comparison to CD5- B cells.** B cells were isolated from four donors and six CLL patient samples by CD19+selection. Control B cells were stained with anti-human CD5 and CD19 antibodies, FACS sorted for CD5-CD19+(CD5-) and CD5+CD19+(CD5+) B cells and miRNA expression was analyzed by RT-PCR. Relative expression of miRNAs in CD5- (square), CD5+control B cells (diamond) and CLL samples (triangle) was normalized to RNU44. Each bar indicates the average expression in each group. *p* value of the analysis calculated by t-test is shown.
(TIF)

Figure S5 **Signature miRNA expression is not significantly altered in CLL cells before and after CD19 positive selection.** Total RNA extracted from PBMC or purified CD19+CLL cells from three CLL patient samples with high white blood cell counts (>50×10^3^/mL blood) (CLL) were analyzed by miRNA-specific RT-PCR. The relative expression of miR-26a, miR-29a, miR-29b, miR-150, miR-155, miR-181a, miR-181b, and miR-223 are shown.
(TIF)

Figure S6 **Inhibition of miR-155 expression by MEK and JNK inhibitors.** A. Relative expression of miR-155 in CLL cells treated with MEK (PD98059; PD) and JNK (SP600125; SP) inhibitors. B. Relative expression of miR-155 in CpG-activated B cell treated with PD98059 and SP600125 inhibitors. Cells were cultured with DMSO as a control (DMSO).
(TIF)

Figure S7 **miRNA expression distinguishes unstimulated B cells from activated B cells.** A. anti-IgM was used to activate B cells for 24 or 48 hrs and miRNA-specific RT-PCRs was performed on control B cells before and after activation. B. The same analysis as in (A) by CD40L for 24 hr or 48 hrs. Co-culturing with the 3T3 feeder cells as the control. C. The same analysis as in (A) after LPS, CpG or poly(I∶C) activation for 24 hr or 48 hrs. Error bars indicate the standard deviation of duplicates.
(TIF)

Table S1 **Summary of clinical parameters of CLL patients samples used in these studies.**
(DOC)

Table S2 **miRNAs tested in FlexmiR v2 expression profiling.**
(DOC)

Table S3 **Cell surface marker expression is compared among unactivated donor B cells, anti-IgM-activated donor B cells, and CLL cells.** FACS analysis was performed using FITC-labeled different surface marker antibodies. 16 different CLL samples (L#), 5 donor control B cell samples (CB) and 3 activated B cell samples by anti-IgM F(ab')_2_ are shown.
(DOC)

Table S4 **miRNAs correlated with ZAP70 and IgV_H_ status.** Wilcoxon rank sum tests were used for whether there were any differences on miRNA expression pattern between patients with different clinical features such as (S4A) ZAP70 status, and (S4B) IgV_H_ status. *p*-value and *q*-value for each miRNA are shown. N: patient number.
(DOC)

Table S5 **CLL-specific miRNA signatures identified by different groups.**
(DOC)

Methods S1 (DOC)
