# Supplementary figures and images for: MicroRNA Expression Profiling Identifies Activated B Cell Status in Chronic Lymphocytic Leukemia Cells
Source: PLoS One. 2011 Mar 8;6(3):e16956. doi: 10.1371/journal.pone.0016956 (PMC3050979; doi:10.1371/journal.pone.0016956)

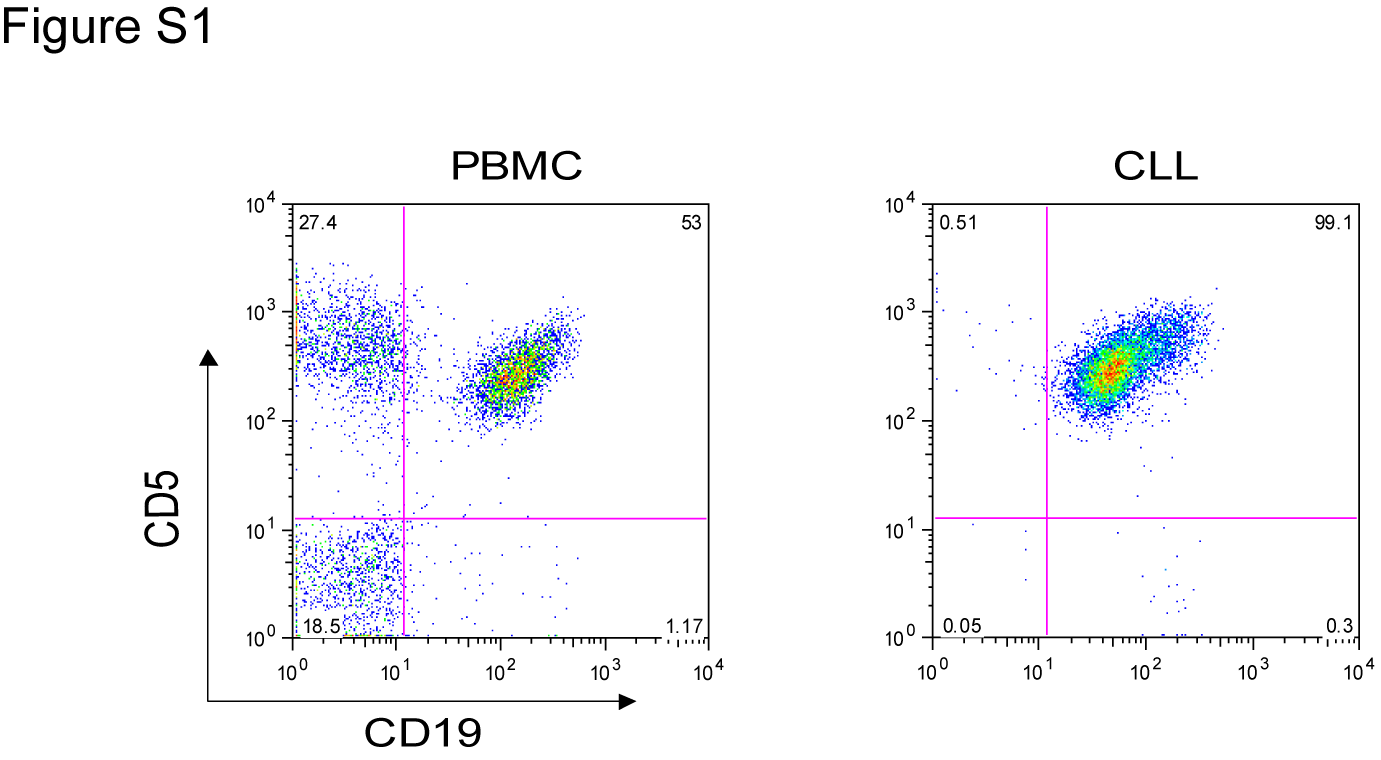

Supplement: Figure S1 — Purity of CLL sample after CD19 positive selection. FACS analysis was performed on PBMCs (PBMC) and purified CLL cells (CLL) using anti-human CD19 and anti-human CD5 antibodies. The purity of the CLL cells was shown as the percentage number on the CD5+CD19+population. (TIF) [file pone.0016956.s001.tif]

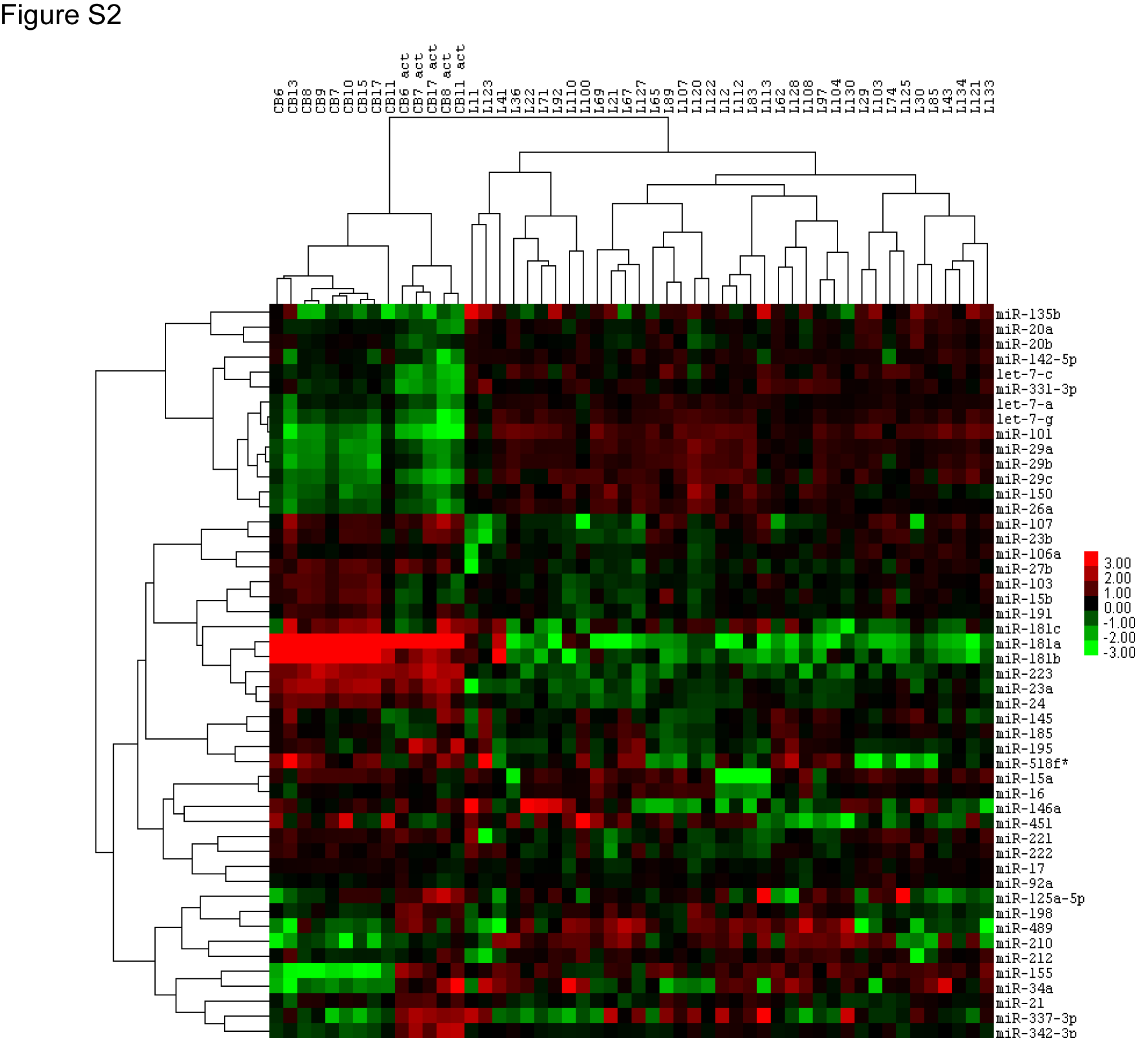

Supplement: Figure S2 — Hierarchical clustering of miRNAs expressed in CLL, control B cell, and activated B cells. Heatmap of miRNA expression across 38 patient-derived CLL samples, 9 donor B cell samples, and 6 CpG activated donor B cell samples. miRNA expression is hierarchically clustered on the Y-axis and patient-derived CLL samples or control B cell donors are hierarchically clustered on the X-axis. The relative expression of miRNAs is depicted according to the color scale shown on the right. CB#: donor control B sample; CB# act: donor activated B samples by CpG; L#: CLL samples. (TIF) [file pone.0016956.s002.tif]

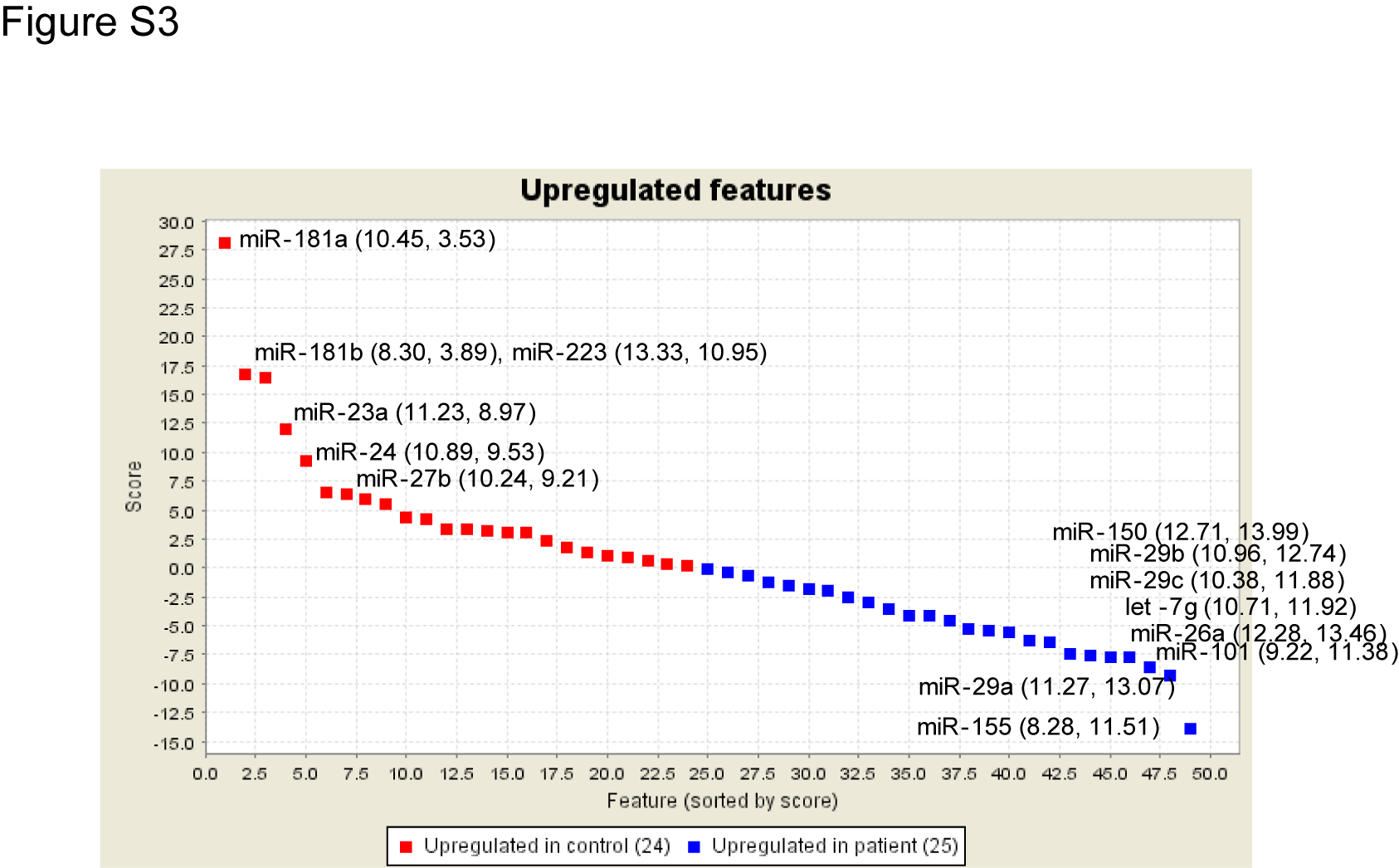

Supplement: Figure S3 — Comparative marker selective view alignment miRNA expression distinguishes miRNA expression between unactivated donor B cells from CLL cells. Comparative marker selective view alignment distinguishes upregulated miRNA expression in 9 control, unactivated B cell sample (Y-axis, red) and in 38 CLL cell patient samples (X-axis, blue). Score (Y-axis) refers to the t-test score indicating the metric correlating gene expression and phenotype. The calculation of t-test score is: (µ1–µ2)/ (s1∧2+s2∧2)∧0.5, where µ1 is the mean of class 1 and µ2 is the mean of class 2, and s1 and s2 are the standard deviation of class 1 and 2. A high score indicates association with the first phenotype (upregulated in control B cells) and a low score indicates association with the second phenotype (upregulated in CLL cells). False Discovery Rate (FDR) is 0.004 for all the miRNAs shown in the diagram. (TIF) [file pone.0016956.s003.tif]

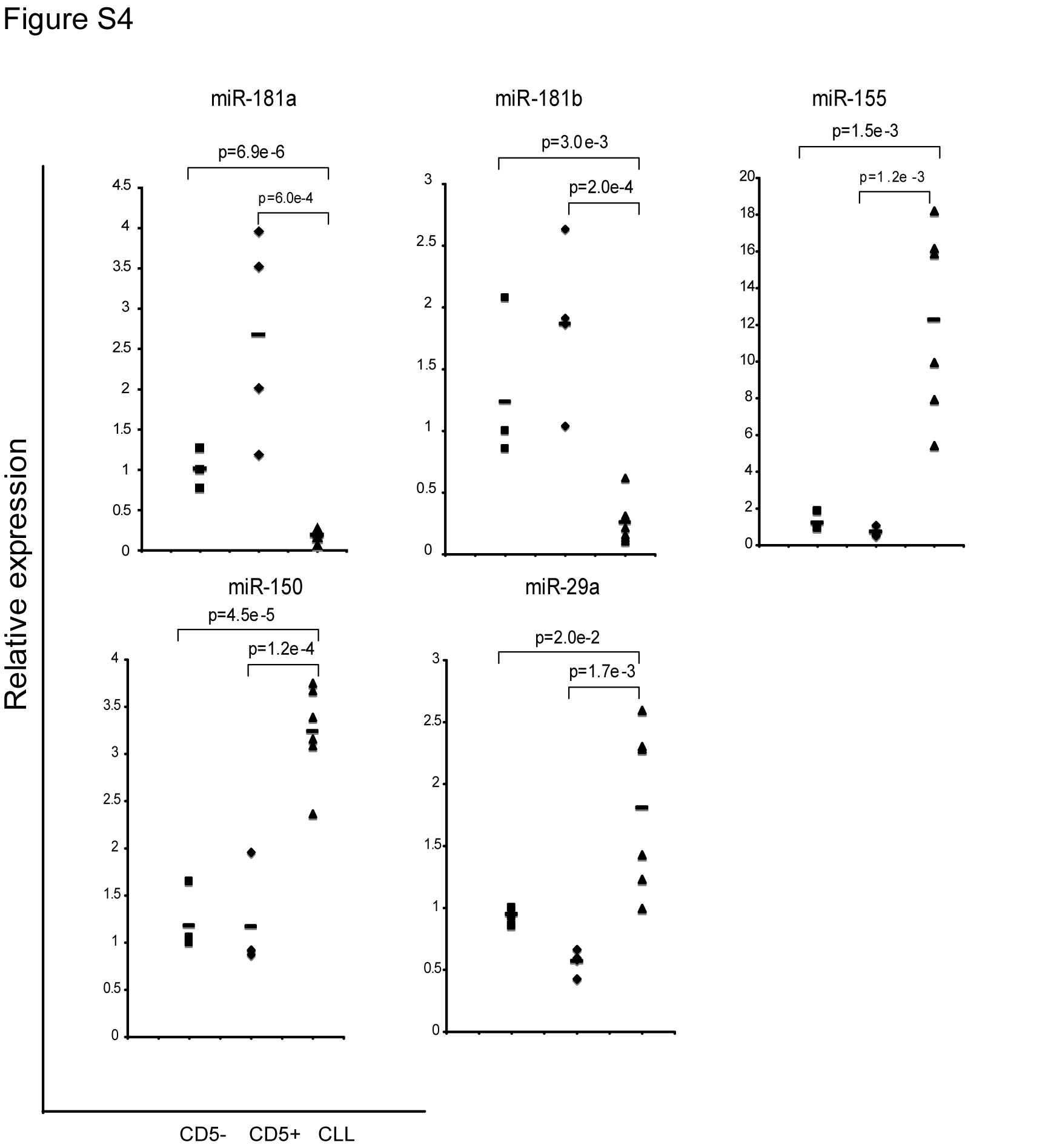

Supplement: Figure S4 — miRNA expression profiling in CD5+B cells in comparison to CD5- B cells. B cells were isolated from four donors and six CLL patient samples by CD19+selection. Control B cells were stained with anti-human CD5 and CD19 antibodies, FACS sorted for CD5-CD19+(CD5-) and CD5+CD19+(CD5+) B cells and miRNA expression was analyzed by RT-PCR. Relative expression of miRNAs in CD5- (square), CD5+control B cells (diamond) and CLL samples (triangle) was normalized to RNU44. Each bar indicates the average expression in each group. p value of the analysis calculated by t-test is shown. (TIF) [file pone.0016956.s004.tif]

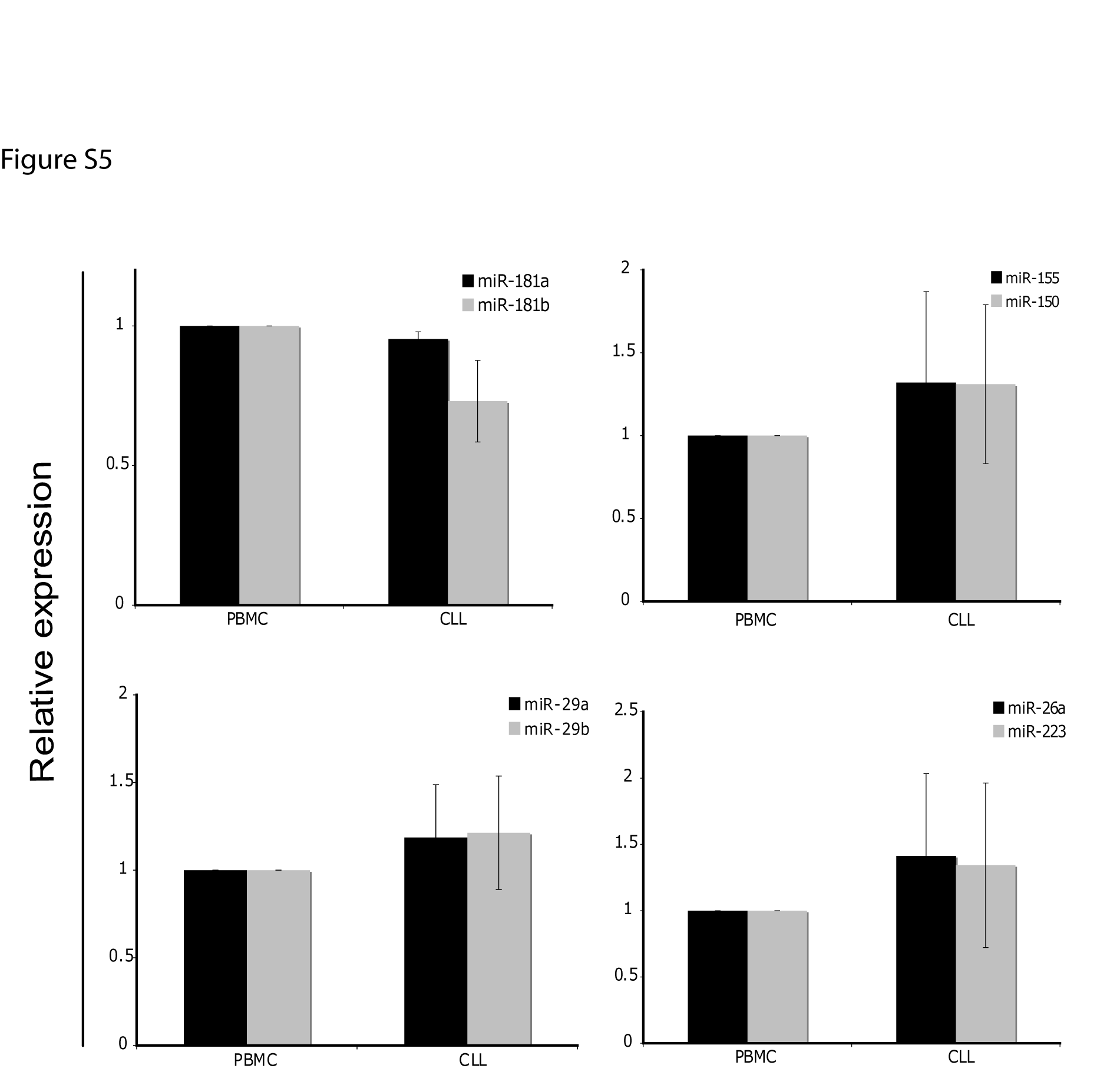

Supplement: Figure S5 — Signature miRNA expression is not significantly altered in CLL cells before and after CD19 positive selection. Total RNA extracted from PBMC or purified CD19+CLL cells from three CLL patient samples with high white blood cell counts (>50×103/mL blood) (CLL) were analyzed by miRNA-specific RT-PCR. The relative expression of miR-26a, miR-29a, miR-29b, miR-150, miR-155, miR-181a, miR-181b, and miR-223 are shown. (TIF) [file pone.0016956.s005.tif]

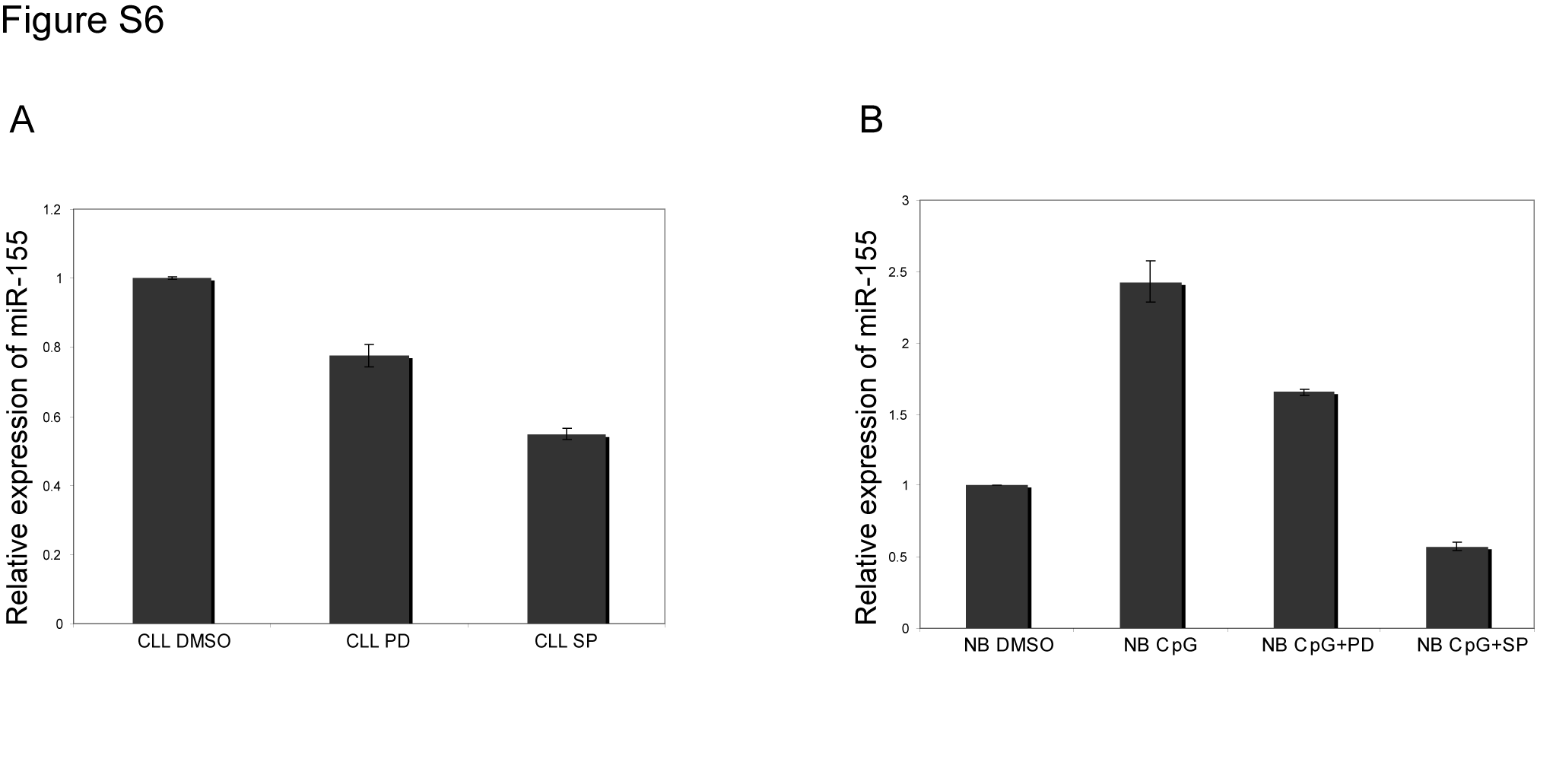

Supplement: Figure S6 — Inhibition of miR-155 expression by MEK and JNK inhibitors. A. Relative expression of miR-155 in CLL cells treated with MEK (PD98059; PD) and JNK (SP600125; SP) inhibitors. B. Relative expression of miR-155 in CpG-activated B cell treated with PD98059 and SP600125 inhibitors. Cells were cultured with DMSO as a control (DMSO). (TIF) [file pone.0016956.s006.tif]

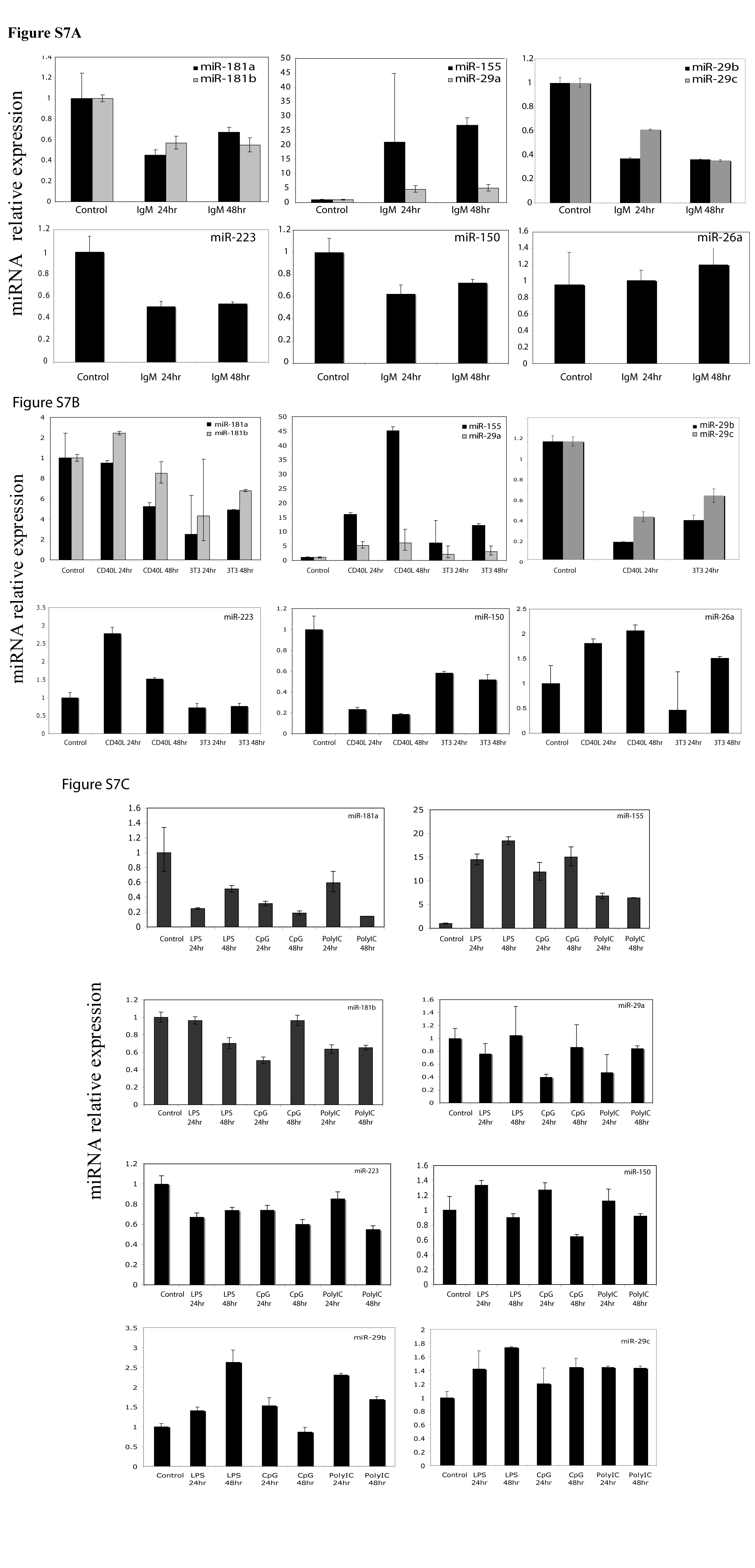

Supplement: Figure S7 — miRNA expression distinguishes unstimulated B cells from activated B cells. A. anti-IgM was used to activate B cells for 24 or 48 hrs and miRNA-specific RT-PCRs was performed on control B cells before and after activation. B. The same analysis as in (A) by CD40L for 24 hr or 48 hrs. Co-culturing with the 3T3 feeder cells as the control. C. The same analysis as in (A) after LPS, CpG or poly(I∶C) activation for 24 hr or 48 hrs. Error bars indicate the standard deviation of duplicates. (TIF) [file pone.0016956.s007.tif]
