# Supplementary material for: MicroRNA Expression Profiling Identifies Activated B Cell Status in Chronic Lymphocytic Leukemia Cells
Source: PLoS One. 2011 Mar 8;6(3):e16956. doi: 10.1371/journal.pone.0016956 (PMC3050979; doi:10.1371/journal.pone.0016956)
Supplement: Table S1 — Summary of clinical parameters of CLL patients samples used in these studies. (DOC) [file pone.0016956.s008.doc]

**Table S1. Summary of clinical parameters of CLL patients samples used in these studies.**

| CLL sample | Age | Sex | Treatment | IgV_H_ mutation | ZAP +/- | Trisomy 12 | 13q deletion (heterozygous) | 13q deletion (homozygous) | 11q deletion | 17p deletion | WBC, 10^3^/µl |
| --- | --- | --- | --- | --- | --- | --- | --- | --- | --- | --- | --- |
| L11 | 50 | M | no | mutated | neg | 50 | 10 | 0 | 0 | 0 | 110.5 |
| L12 | 52 | M | no | mutated | neg | 0 | 0 | 27 | 0 | 0 | 64.8 |
| L21 | 70 | M | no | unmutated | pos | 0 | 83 | 0 | 0 | 0 | 84.1 |
| L22 | 63 | F | no | unmutated | pos | 0 | 96 | 0 | 0 | 0 | 56.4 |
| L29 | 61 | M | no | mutated | neg | 0 | 0 | 0 | 0 | 0 | 148.1 |
| L30 | 60 | F | no | mutated | neg | 0 | 78 | 0 | 5 | 10 | 47.6 |
| L36 | 79 | F | no | mutated | neg | 0 | 47 | 0 | unknown | 12 | 178.9 |
| L41 | 53 | F | no | mutated | neg | 0 | 72 | 0 | 0 | 0 | 205.9 |
| L43 | 60 | M | no | mutated | neg | 0 | 75 | 15 | 0 | 0 | 160.6 |
| L62 | 40 | M | no | unmutated | pos | 0 | 66 | 0 | 0 | 0 | 159.9 |
| L65 | 70 | M | no | unmutated | pos | 0 | 0 | 10 | 0 | 0 | 71.9 |
| L67 | 65 | F | yes | unknown | pos | 0 | 73 | 0 | 86 | 0 | 201 |
| L69 | 43 | F | no | mutated | neg | 3 | 3 | 0 | 0 | 0 | 114.5 |
| L71 | 63 | M | no | mutated | pos | 0 | 0 | 83 | 0 | 0 | 112.5 |
| L74 | 57 | F | no | mutated | neg | 0 | 0 | 71 | 0 | 0 | 93.8 |
| L83 | 60 | M | no | mutated | pos | 0 | 0 | 50 | 0 | 0 | 22.6 |
| L85 | 59 | F | no | unmutated | pos | 0 | 0 | 0 | 0 | 0 | 24.9 |
| L89 | 76 | F | yes | unmutated | pos | 0 | 22 | 0 | 0 | 34 | 214.2 |
| L92 | 61 | M | no | unknown | neg | 0 | 0 | 71 | 0 | 0 | 124.4 |
| L97 | 75 | M | no | mutated | neg | 0 | 30 | 48 | 0 | 0 | 25.1 |
| L100 | 79 | F | no | unmutated | neg | 36 | 5 | 0 | 0 | 0 | 16.4 |
| L103 | 61 | M | no | unknown | neg | 0 | 11 | 0 | 89 | 0 | 116.4 |
| L104 | 65 | M | no | unknown | unknown | 0 | 21 | 0 | 0 | 0 | 213.8 |
| L107 | 62 | F | no | unmutated | pos | 0 | 39 | 0 | 0 | 0 | 78.5 |
| L108 | 67 | F | no | unmutated | pos | 0 | 0 | 0 | 0 | 0 | 15.7 |
| L110 | 87 | M | no | mutated | pos | 24 | 0 | 0 | 0 | 0 | 16.3 |
| L112 | 71 | F | no | mutated | neg | 0 | 0 | 61 | 0 | 0 | 17.1 |
| L113 | 54 | M | no | unmutated | neg | unknown | unknown | unknown | unknown | unknown | 23.8 |
| L120 | 44 | M | no | unknown | unknown | 0 | 77 | 0 | 0 | 0 | 84.1 |
| L121 | 66 | F | no | mutated | neg | 1 | 0 | 61 | 2 | 0 | 57.6 |
| L122 | 54 | F | no | mutated | neg | 0 | 94 | 0 | 0 | 0 | 265.6 |
| L123 | 66 | M | yes | unmutated | pos | 4 of 20 metaphases contained trisomy 12 and translocation t(14;18) | 0 | 0 | 0 | 0 | 61.6 |
| L125 | 52 | F | no | unmutated | neg | 0 | 24 | 0 | 89 | 0 | 117.8 |
| L127 | 50 | F | no | unmutated | neg | 26 | 60 | 0 | 0 | 0 | 57.8 |
| L128 | 77 | F | no | mutated | neg | 0 | 0 | 50 | 0 | 0 | 44.5 |
| L130 | 60 | M | no | unmutated | pos | 0 | 8 | unknown | unknown | 0 | 52.4 |
| L133 | 50 | F | no | unknown | neg | 0 | 0 | 0 | 0 | 0 | 17.2 |
| L134 | 78 | F | no | unknown | neg | 0 | 36 | 0 | 0 | 0 | 69.6 |
