# Supplementary material for: MicroRNA Expression Profiling Identifies Activated B Cell Status in Chronic Lymphocytic Leukemia Cells
Source: PLoS One. 2011 Mar 8;6(3):e16956. doi: 10.1371/journal.pone.0016956 (PMC3050979; doi:10.1371/journal.pone.0016956)
Supplement: Table S2 — miRNAs tested in FlexmiR v2 expression profiling. (DOC) [file pone.0016956.s009.doc]

**Table S2. miRNAs tested in FlexmiR v2 expression profiling.**

| miRNA list | |
| --- | --- |
| let -7a | miR-135b |
| let-7c | miR-142-5p |
| let-7g | miR-145 |
| miR-15a | miR-146a |
| miR-15b | miR-146b-5p |
| miR-16 | miR-150 |
| miR-17 | miR-155 |
| miR-20a | miR-181a |
| miR-20b | miR-181b |
| miR-21 | miR-181c |
| miR-23a | miR-185 |
| miR-23b | miR-191 |
| miR-24-1 | miR-195 |
| miR-26a | miR-198 |
| miR-27b | miR-210 |
| miR-29a | miR-212 |
| miR-29b | miR-221 |
| miR-29c | miR-222 |
| miR-34a | miR-223 |
| miR-92 | miR-331-3p |
| miR-101 | miR-337-3p |
| miR-103 | miR-342-3p |
| miR-106a | miR-451 |
| miR-107 | miR-489 |
| miR-125a-5p | miR-518f* |
