# Supplementary material for: MicroRNA Expression Profiling Identifies Activated B Cell Status in Chronic Lymphocytic Leukemia Cells
Source: PLoS One. 2011 Mar 8;6(3):e16956. doi: 10.1371/journal.pone.0016956 (PMC3050979; doi:10.1371/journal.pone.0016956)
Supplement: Table S3 — Cell surface marker expression is compared among unactivated donor B cells, anti-IgM-activated donor B cells, and CLL cells. FACS analysis was performed using FITC-labeled different surface marker antibodies. 16 different CLL samples (L#), 5 donor control B cell samples (CB) and 3 activated B cell samples by anti-IgM F(ab')2 are shown. (DOC) [file pone.0016956.s010.doc]

**Table S3. Cell surface marker expression is compared among unactivated donor B cells, anti-IgM-activated donor B cells, and CLL cells**.

| Samples | Sample Name | %CD86 | %CD80 | %CD69 |
| --- | --- | --- | --- | --- |
| CLL samples | L12 | 70.6 | 79 | 24.1 |
|  | L16 | 77.4 | 90.7 | 52 |
|  | L22 | 34.7 | 68.7 | 69.6 |
|  | L29 | 41.3 | 44.3 | 32.8 |
|  | L30 | 75.4 | 82.3 | 40.1 |
|  | L35 | 68.9 | 72 | 53.2 |
|  | L39 | 86.2 | 53.2 | 36.1 |
|  | L40 | 59.2 | 40.3 | 30.8 |
|  | L41 | 75.4 | 85.5 | 80.8 |
|  | L60 | 83.9 | 80 | 91.1 |
|  | L62 | 55.9 | 45.4 | 32.9 |
|  | L63 | 66.9 | 64 | 73.2 |
|  | L64 | 15.6 | 61.1 | 37.3 |
|  | L65 | 48.5 | 82.4 | 26.5 |
|  | L66 | 71.3 | 70.5 | 68.5 |
|  | L67 | 23 | 75.5 | 79.8 |
| Donor B sample | CB1 | 59.2 | 34.4 | 43.1 |
|  | CB2 | 50.3 | 29.1 | 27.4 |
|  | CB11 | 77 | 37.4 | 19.9 |
|  | CB14 | 44.8 | 17.7 | 2.77 |
|  | CB19 | 45.4 | 10.5 | 11.5 |
| CB2 activation | Anti-IgM | 83 | 61.1 | 62.3 |
| CB11 activation | Anti-IgM | 87.4 | 60.9 | 55 |
| CB19 activation | Anti-IgM | 82.5 | 23 | 56.2 |

FACS analysis was performed using FITC-labeled different surface marker antibodies. 16 different CLL samples (L#), 5 donor control B cell samples (CB) and 3 activated B cell samples by anti-IgM F(ab’)_2_ are shown.
