# Supplementary material for: MicroRNA Expression Profiling Identifies Activated B Cell Status in Chronic Lymphocytic Leukemia Cells
Source: PLoS One. 2011 Mar 8;6(3):e16956. doi: 10.1371/journal.pone.0016956 (PMC3050979; doi:10.1371/journal.pone.0016956)
Supplement: Table S4 — miRNAs correlated with ZAP70 and IgVH status. Wilcoxon rank sum tests were used for whether there were any differences on miRNA expression pattern between patients with different clinical features such as (S4A) ZAP70 status, and (S4B) IgVH status. p-value and q-value for each miRNA are shown. N: patient number. (DOC) [file pone.0016956.s011.doc]

**Table S4A.** **miRNAs correlated with ZAP70 status.**

| miRNAs | ZAP70 | | | |  |  |
| --- | --- | --- | --- | --- | --- | --- |
|  | Negative | | Positive | | *p* | *q* |
|  | N | Median (Min, Max) | N | Median (Min, Max) |  |  |
| miR-150 | 22 | 13.67 (13.16, 14.89) | 14 | 14.05 (13.64, 15.05) | 0.009 | 0.09 |
| miR-223 | 22 | 11.28 (10.08, 11.98) | 14 | 10.81 (9.47, 11.23) | 0.005 | 0.08 |
| miR-29c | 22 | 12.13 (10.99, 12.53) | 14 | 11.62 (10.90, 12.13) | 0.006 | 0.09 |
| miR-92a | 22 | 11.10 (10.81, 11.72) | 14 | 10.95 (10.40, 11.24) | 0.004 | 0.08 |

**Table S4B.** **miRNAs correlated with IgV_H_ status.**

| miRNAs | IgV_H_ mutation | | | |  |  |
| --- | --- | --- | --- | --- | --- | --- |
|  | Mutated | | Unmutated | | *p* | *q* |
|  | N | Median (Min, Max) | N | Median (Min, Max) |  |  |
| let-7g | 17 | 12.01 (11.54, 12.33) | 14 | 11.79 (11.22, 12.34) | 0.005 | 0.07 |
| miR-223 | 17 | 11.29 (10.62,11.98) | 14 | 10.65 (9.47, 11.07) | 0.0005 | 0.02 |
| miR-29c | 17 | 12.20 (11.25, 12.53) | 14 | 11.49 (10.90, 12.25) | 0.004 | 0.06 |

Wilcoxon rank sum tests were used for whether there were any differences on miRNA expression pattern between patients with different clinical features such as (S4A) ZAP70 status, and (S4B) IgV_H_ status. *p*-value and *q*-value for each miRNA are shown. N: patient number.
