# Supplementary material for: MicroRNA Expression Profiling Identifies Activated B Cell Status in Chronic Lymphocytic Leukemia Cells
Source: PLoS One. 2011 Mar 8;6(3):e16956. doi: 10.1371/journal.pone.0016956 (PMC3050979; doi:10.1371/journal.pone.0016956)
Supplement: Table S5 — CLL-specific miRNA signatures identified by different groups. (DOC) [file pone.0016956.s012.doc]

**Table S5. CLL-specific** miRNA signatures identified by different groups.

| signature | Li S et al | Valerio F et al | Zanette DL et al | Calin GA et al* |
| --- | --- | --- | --- | --- |
| Reduced | miR-181a  miR-181b  miR-223  miR-23a  miR-24  miR-27b | miR-92  miR-222 | miR-135b  miR-199s  miR-142-5p  miR-185  miR-181c | miR-223  miR-29a-2  miR-29b-2  miR-29c |
| Elevated | miR-155  miR-150  miR-29a  miR-29b  miR-29c  miR-101  miR-26a  let-7g | miR-21  miR-101  miR-150  miR-155 | miR-331  miR-29a  miR-195  miR-34a  miR-29c | miR-15a  miR-195  miR-221  miR-23b  miR-155  miR-24-1  miR-146  miR-16-1  miR-16-2 |

***** Calin et al examined only CLL patients with ZAP70^+^ and IgV_H_ unmutated status.
