# Supplementary material for: MicroRNA Expression Profiling Identifies Activated B Cell Status in Chronic Lymphocytic Leukemia Cells
Source: PLoS One. 2011 Mar 8;6(3):e16956. doi: 10.1371/journal.pone.0016956 (PMC3050979; doi:10.1371/journal.pone.0016956)
Supplement: Methods S1 — (DOC) [file pone.0016956.s013.doc]

## Methods S1

**Analysis of ZAP-70 and sequence analysis of IgV_H_**

ZAP-70 was assessed by flow cytometry, and the expressed IgV_H_ was determined by reverse transcription polymerase chain reaction (RT-PCR) and enzyme-linked immunosorbent assay as previously described [1]. The cDNA was amplified by PCR, purified, sequenced and % homology was determined relative to the closest germline match as previously described [1]. ZAP-70 expression (positive defined as >20%) and IgV_H_ homology (unmutated defined as greater than or equal to 98% homology to the closest germline match) were determined by the CLL Research Consortium tissue core.

**Genomic aberrations by fluorescence in situ hybridization**

Cytogenetics were evaluated by FISH for the most common abnormalities (del 11q, del 13q, del 17p, and trisomy 12) [2] according to the standard clinical practice at the Brigham and Women’s Hospital Cytogenetics lab, the number reported for cytogenetics in Table S1 is percent abnormal by FISH.

**Supplementary References**

1. Rassenti LZ, Huynh L, Toy TL, Chen L, Keating MJ, et al. (2004) ZAP-70 compared with immunoglobulin heavy-chain gene mutation status as a predictor of disease progression in chronic lymphocytic leukemia. N Engl J Med 351: 893-901.

2. Dohner H, Stilgenbauer S, Benner A, Leupolt E, Krober A, et al. (2000) Genomic aberrations and survival in chronic lymphocytic leukemia. N Engl J Med 343: 1910-1916.
